# Epigenetic regulation of VENTXP1 suppresses tumor proliferation via miR-205-5p/ANKRD2/NF-kB signaling in head and neck squamous cell carcinoma

**DOI:** 10.1038/s41419-020-03057-w

**Published:** 2020-10-09

**Authors:** Li Ming Zhang, Li Xin Su, Jing Zhou Hu, De Ming Wang, Hou Yu Ju, Xiao Li, Yi Feng Han, Wei Ya Xia, Wei Guo, Guo Xin Ren, Xin Dong Fan

**Affiliations:** 1 Department of Interventional Therapy, Shanghai Ninth People’s Hospital, Shanghai Jiao Tong University School of Medicine, Shanghai, China; 2 Shanghai Key Laboratory of Stomatology & Shanghai Research Institute of Stomatology; National Clinical Research Centre for Oral Diseases, Shanghai, China; 3 Department of Oral and Maxillofacial-Head and Neck Oncology, Shanghai Ninth People’s Hospital, Shanghai Jiao Tong University School of Medicine, Shanghai, China; 4 Department of Molecular and Cellular Oncology, The University of Texas MD Anderson Cancer Center, 1515 Holcombe Blvd, Houston, TX 77030 USA

**Keywords:** Oral cancer, Oncogenesis

## Abstract

An increasing number of studies have shown that long noncoding RNAs (lncRNAs) play important roles in tumor development and progression. However, their involvement in head and neck squamous cell carcinoma (HNSCC) remains largely unknown. Epigenetic regulation is one major mechanism utilized by cancer cells to control lncRNA expression. We identified that lncRNA VENTXP1 was epigenetically silenced in multiple cancer types, and its lower expression was correlated with poorer survival in HNSCC patients. Through in silico analysis and experimental validation, we identified miR-205-5p and its direct interacting partner of VENTXP1, which regulates HNSCC cell proliferation and tumorigenicity. Using RNA-seq and differential gene expression analysis, we further identified ANKRD2 as a miR-205-5p target, which plays an essential role in modulating NF-kB signaling. These findings suggest that VENTXP1 inhibits tumor growth via suppressing miR-205-5p/ANKRD2-mediated NF-kB signaling in HNSCC. Thus, pharmaceutical targeting of DNA methylation to restore VENTXP1 expression might constitute a therapeutic strategy for HNSCC.

## Introduction

Head and neck squamous cell carcinoma (HNSCC) is the sixth most common malignant tumor worldwide^1^. Despite progresses achieved in therapies, such as using nivolumab and pembrolizumab to target the programmed cell death protein 1 (PD-1) in advanced HNSCC, the overall 5-year survival rate for patients with HNSCC remains unsatisfactory, which is below 50%. Moreover, many patients eventually developed metastatic recurrence^2^. Several predisposing factors, including smoking and alcohol consumption, have been identified, but the mechanism of HNSCC carcinogenesis remains elusive^2^. Thus, understanding the mechanisms underlying HNSCC progression is essential for improving HNSCC treatment.

The results from human genome sequencing projects indicate that protein-coding sequences occupy less than 2% of the human genome^3^. Long noncoding RNAs (lncRNAs) refer to a class of RNA transcripts longer than 200 nucleotides with limited protein-coding potential. Aberrant expression of lncRNAs has been associated with the occurrence and development of various types of cancers^4^, including HNSCC. lncRNA plays a role in regulating transcription levels through interacting with transcription machinery and regulators. In addition, lncRNA can also act as “sponges” to titrate microRNAs (miRNAs), thus participating in post-transcriptional processing^5–8^. For example, MALAT1 has been reported to be upregulated in lung^9^ and gastric cancers^10^. Overexpression of MALAT1 increases cell proliferation, induces cell migration, and is correlated with tumor metastasis^11^. Another lncRNA, HOTAIR, functions as an oncogene in multiple cancer types, which also promotes exosome secretion from liver cancer cells^12^. The lncRNA ANRIL has been reported to regulate the proliferation of HNSCC cells via the miR-125a-3p/FGFR1/MAPK signaling axis^13^.

Epigenetic regulation is a new mechanism regulating lncRNA expression and tissue specificity^14–16^. Dysregulation of DNA methylation in cancer is a hallmark of tumorigenesis^17,18^. However, epigenetic changes in genes encoding lncRNAs and the effects of such changes in cancers remain poorly characterized.

In our present study, we integrated epigenetic and multidimensional genomic data from the Cancer Genome Atlas (TCGA) and Cancer Cell Line Encyclopedia (CCLE) databases^19^. We characterized the DNA methylation landscape of lncRNA genes across four cancer types, and found that methylation of the gene enocoding lncRNA VENTXP1 was recurrently altered in tumors. By further integrating these data with the clinical data and the results from mechanism study, we proposed an oncogenic role of lncRNA VENTXP1 in tumor proliferation and metastasis.

## Results

### Identification of epigenetically regulated lncRNAs in four types of cancers

We first characterized the lncRNA DNA methylation pattern in four types of cancers (breast, liver, lung, and head and neck cancers) by comparing the DNA methylation profiles of lncRNA promoters in tumors with normal tissues using data from the TCGA projects. Previous studies have established the CpG island hypermethylation phenotype (CIMP) as a hallmark in many cancer types; here we aimed to focus on studying hypermethylated lncRNAs.

**Fig. 1:**
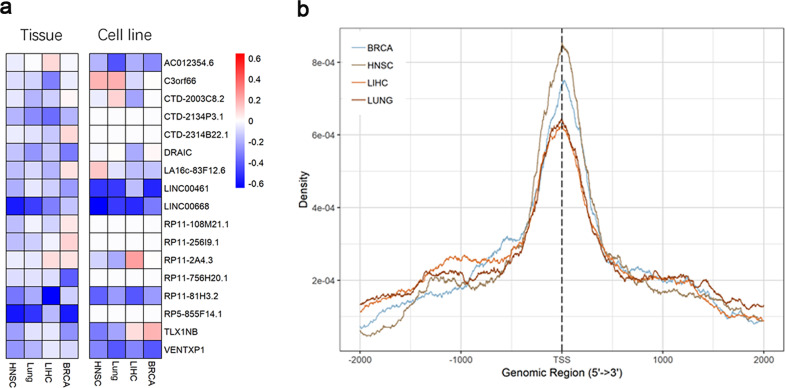
Epigenetic landscape of lncRNAs in cancers. **a** Fold changes in DNA methylation of significant epigenetically silenced lncRNAs in four cancer types. **b** Distribution of the differential DNA methylation within the region ±3 kb of epigenetically silenced lncRNA gene TSS in four cancer types.

To determine whether the expression of lncRNA is regulated by the status of DNA methylation in its promoters, we retrieved the lncRNA expression data from MiTranscriptome, which summarizes cancer-associated lncRNA transcripts. Our study was restricted to four cancer types in TCGA for which both DNA methylation and lncRNA expression data were available. We identified the lncRNAs that were epigenetically activated or silenced in tumors by comparing their DNA methylation status with normal tissues. We found 1798 lncRNA genes across four cancer types, including 987 epigenetically activated and 811 epigenetically silenced lncRNAs that showed epigenetic alterations in at least one cancer type. To further validate the correlation between methylation status of the lncRNA and its expression in cancers, we integrated cell line RNA-seq and DNA methylation profiles for all four types of cancers in CCLE^19^. The most frequently epigenetically silenced lncRNAs are shown in Fig. 1a. Moreover, methylation intensity of epigenetically silenced lncRNAs is enriched near TSS (Fig. 1b), which is consistent with the notion that hypermethylation of a gene promoter suppresses its transcription.

### Epigenetic silencing of VENTXP1 correlated with favorable survival in HNSCC

**Fig. 2:**
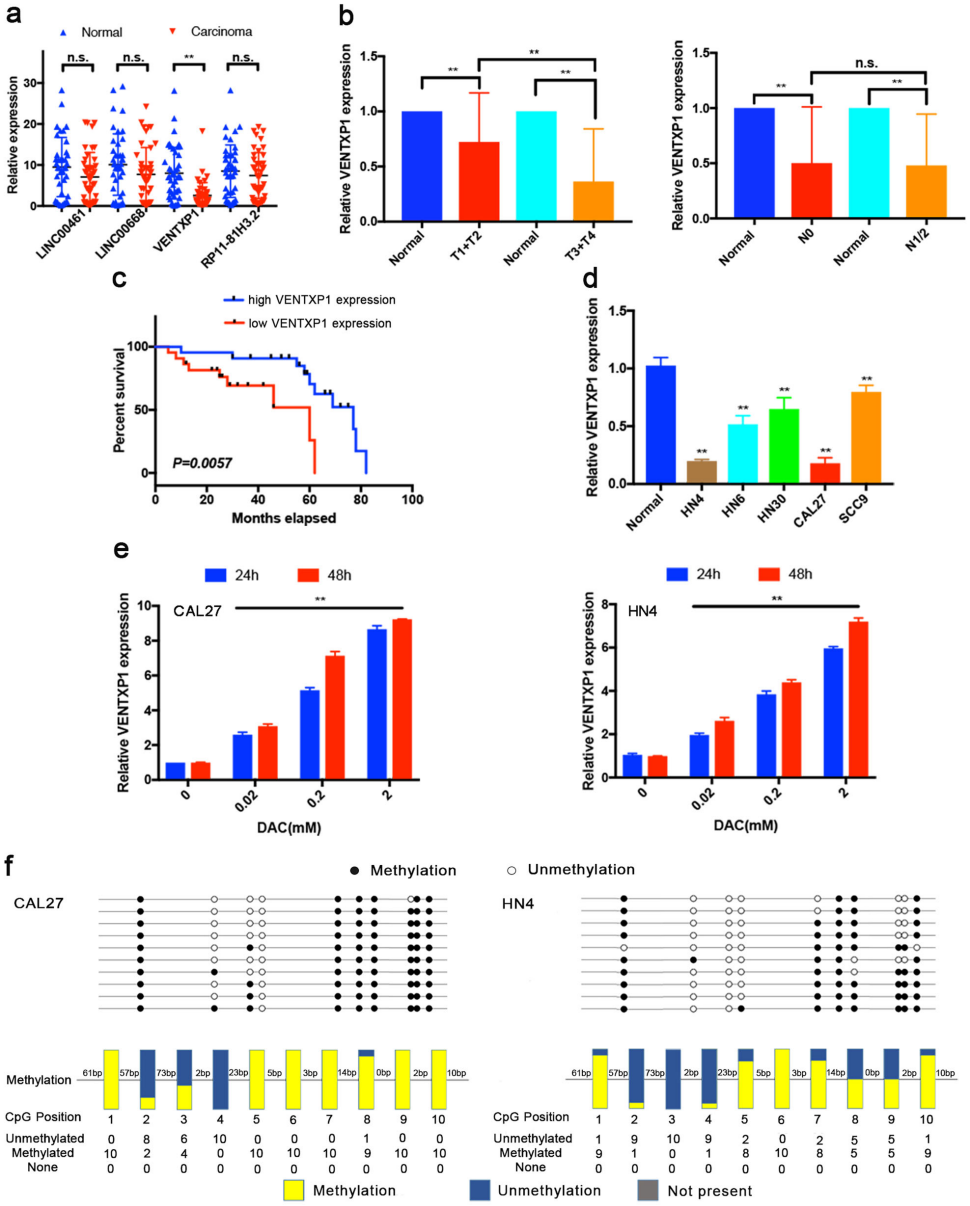
VENTXP1 is associated with favorable survival and epigenetically silenced in head and neck squamous cell carcinoma (HNSCC). **a** Expression levels of four epigenetically silenced lncRNAs in HNSCC tissues and matched normal tissues (*n* = 44), normalized by actin expression. Differences in expression were analyzed with paired samples *t* tests. **b** Correlations between VENTXP1 expression and tumor stage. The expression of VENTXP1 in T1/2 versus T3/4-stage tumors (left) and N0 versus N1/2-stage tumors (right) was normalized to that in the corresponding paired normal tissues. **c** HNSCC patients with high VENTXP1 expression in tumor tissue showed significantly better overall survival (OS) than those with low VENTXP1 expression. **d** Expression levels of VENTXP1 in five HNSCC cell lines (HN4, HN6, HN30, CAL27, and SCC9) and normal oral primary keratinocytes (titled “Normal”). The expression of VENTXP1 was normalized to that in Normal cells. The relative expression fold changes in mRNA expression were calculated by the 2^−ΔΔCt^ method. **e** QRT-PCR analysis of VENTXP1 expression in HN4 and CAL27 cells treated with decitabine (DAC). **f** Bisulfite genomic sequencing was performed to examine the methylation status of the CpG islands in the promoter region of VENTXP1 in HN4 and CAL27 cells. The horizontal lines in the box plots indicate the medians, the boxes indicate the interquartile ranges, and the whiskers indicate the 2.5th and 97.5th percentiles. **P < 0.05; n.s. not significant.

In silico analysis indicated that four lncRNAs, LINC00461, LINC00668, VENTXP1, and RP11-81H3.2 were most significantly silenced in both tumors and cancer cell lines (Fig. 1a). We further examined the expression of these lncRNAs using qRT-PCR in tumors from 44 HNSCC patients along with paired adjacent normal tissues (Fig. 2a). This revealed that only expression of VENTXP1 was significantly lower in carcinoma than in normal tissues. Therefore, we decided to focus our study on VENTXP1 in HNSCC.

When combined with clinical parameters from HNSCC tumors, we observed a further reduction of VENTXP1 expression in stage T3 and T4 versus that in stage T1 and T2 tumors (Fig. 2b). However, this is not the case when similar analysis was done on tumors with low and high lymph- node dissemination and metastasis status (Fig. 2b). To further elucidate the clinical significance of VENTXP1 in HNSCC, we investigated the potential associations between VENTXP1 expression and patient clinicopathological features (Table 1). Lower expression of VENTXP1 was found to be associated with a more advanced T stage, higher clinical stage, and smoking. Patients with lower VENTXP1 expression in HNSCC tissues, as stratified by median VENTXP1 expression, showed significantly poorer overall survival (Fig. 2c). To facilitate mechanistic study, we also examined VENTXP1 expression in a panel of HNSCC cell lines. This showed that VENTXP1 expression was decreased in all HNSCC cell lines when compared with normal oral primary keratinocytes, which was consistent with the results from patient biopsies (Fig. 2d).

**Table 1:** Clinicopathological features correlated with lncRNA VENTXP1 expression in HNSCC patients.

		Expression of lncRNA *VENTXP1*		
Characteristics	Number of cases (%)	Low (%)	High (%)	*P* value
*Gender*				
Male	25 (56.8)	13 (52.0)	12 (48.0)	0.761
Female	19 (43.2)	9 (47.4)	10 (52.6)	
*Ages, years*				
≤ 59	22 (50)	10 (45.5)	12 (54.5)	0.546
>59	22 (50)	12 (54.5)	10 (45.4)	
*T stage, TNM*				
T1 + T2	32 (72.7)	11 (34.4)	21 (65.6)	0.001^**^
T3 + T4	12 (27.3)	11 (91.7)	1 (8.3)	
*Clinical stage*				
I–II	28 (63.6)	9 (32.1)	19 (67.9)	0.002^**^
III–IV	16 (36.4)	13 (81.3)	3 (18.7)	
*Lymph-node metastasis*				
Negative	27 (61.4)	13 (48.1)	14 (51.9)	0.757
Positive	17 (38.6)	9 (52.9)	8 (47.1)	
*Alcohol drinking*				
Absent	27 (61.4)	12 (44.4)	15 (55.6)	0.353
Present	17 (38.6)	10 (58.9)	7 (41.1)	
*Smoking*				
Absent	29 (65.9)	11 (37.9)	18 (62.1)	0.026^**^
Present	15 (34.1)	11 (73.3)	4 (26.7)	
*Location*				
Lips	17 (38.6)	11 (64.7)	6 (35.3)	0.092
Cheek	8 (18.2)	3 (37.5)	5 (62.5)	
Buccal mucosa	13 (29.5)	7 (53.8)	6 (46.2)	
Tongue	6 (13.7)	1 (16.7)	5 (83.3)	

*HNSCC* head and neck squamous cell carcinoma, *TNM* tumor-node metastasis. ^**^P < 0.05.

To confirm whether expression of VENTXP1 is regulated by hypermethylation of its gene promoter, we performed bisulfite sequencing and found that more than 70% of the promoter region of VENTXP1 showed hypermethylation in two HNSCC cells (Fig. 2f). Consistent with this finding, when CAL27 and HN4 cells were treated with decitabine (5-aza-2), a DNA demethylation agent, VENTXP1 expression was induced in a dose- and time-dependent manner in both cell lines (Fig. 2e). Collectively, our results demonstrated that VENTXP1 expression is regulated by its promoter methylation in HNSCC.

### VENTXP1 functions as a putative anti-oncogenic lncRNA by inhibiting HNSCC cell proliferation in vitro and in vivo

**Fig. 3:**
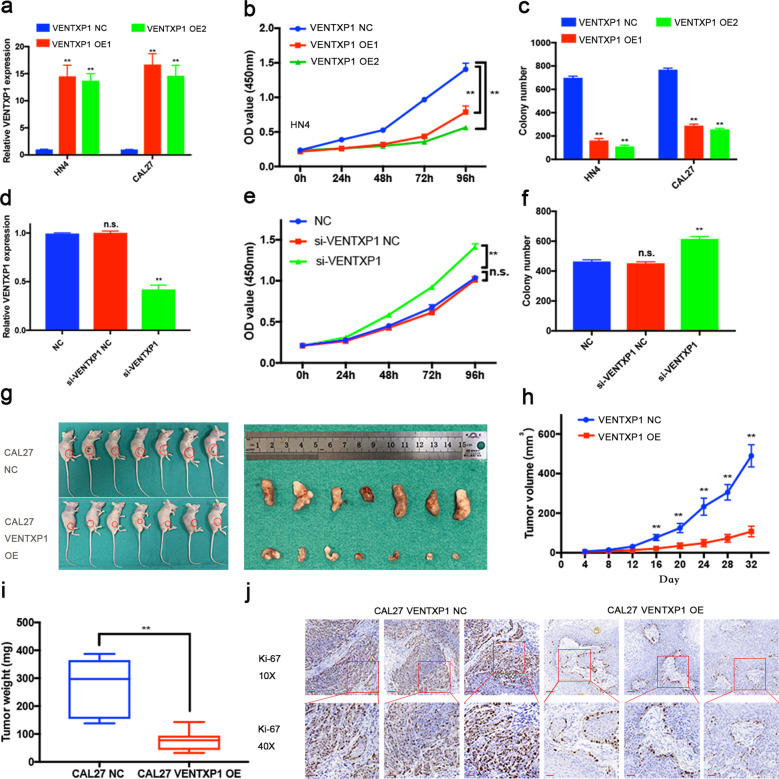
VENTXP1 inhibits the growth of head and neck squamous cell carcinoma (HNSCC) in vitro and in vivo. **a** VENTXP1 expression was measured in HN4 and CAL27 cells via qRT-PCR after transfection with expression plasmids for 48 h. **b** Cell growth viability was measured in VENTXP1-OE (overexpression plasmid)-transfected HN4 cells by CCK-8 assays. **c** Results of colony-formation assays after HN4 and CAL27 cells were transfected with VENTXP1 NC or -OE. **d** VENTXP1 expression was measured in normal oral primary keratinocytes via qRT-PCR after transfection with si-VENTXP1 NC or si-VENTXP1 for 48 h. **e** Cell growth viability was measured in si-VENTXP1-transfected normal oral primary keratinocytes by CCK-8 assays. **f** Results of colony-formation assays after normal oral primary keratinocytes were transfected with si-VENTXP1 NC or si-VENTXP1. **g** Tumors derived from the xenograft model were excised and are shown for each group. **h** Tumor growth curves for mice injected with cells transfected with VENTXP1-OE lentiviral or control vector were analyzed. **i** The weight of tumors excised from mice in the ectopic expression and vector groups was measured and analyzed. **j** Representative images of Ki-67 immunohistochemical staining in each group are shown (bar = 100 μm, 25 μm, respectively). **P < 0.05; n.s. not significant.

To evaluate the functional role of VENTXP1 in HNSCC, we selected CAL27 and HN4 cells because they exhibited the lowest expression levels of VENTXP1 among HNSCC cells (Fig. 2d). We overexpressed VENTXP1 in both cell lines. The overexpression efficiency was verified using qRT-PCR (Fig. 3a). Overexpression of VENTXP1 significantly reduced the viability and proliferation of both HN4 and CAL27 (Fig. 3b and Supplementary Fig. s1a). Similarly, the colony-formation assay results revealed that clonogenic survival was significantly decreased by overexpression of VENTXP1 (Fig. 3c and Supplementary Fig. s1b). In addition, we transfected normal oral primary keratinocytes with control and si-VENTXP1, and verified the knockdown via qRT-PCR (Fig. 3d). Cell -viability and colony-formation assay showed that the viability and clonogenic survival of normal oral primary keratinocytes were enhanced by knocking down of VENTXP1 (Fig. 3e, f and Supplementary Fig. s1c). To assess the tumor-suppressing function of VENTXP1 in vivo, we established a xenograft model of HNSCC. Tumor volumes and weights were decreased in mice harboring the VENTXP1-overexpressing tumors (Fig. 3g–i). In addition, the staining intensity of the proliferation antigen Ki-67 was significantly decreased in the VENTXP1-overexpressing group (Fig. 3j). All these findings demonstrated that lncRNA VENTXP1 played a potential anti-oncogenic role in HNSCC.

### Identification of miRNAs potentially targeted by VENTXP1

**Fig. 4:**
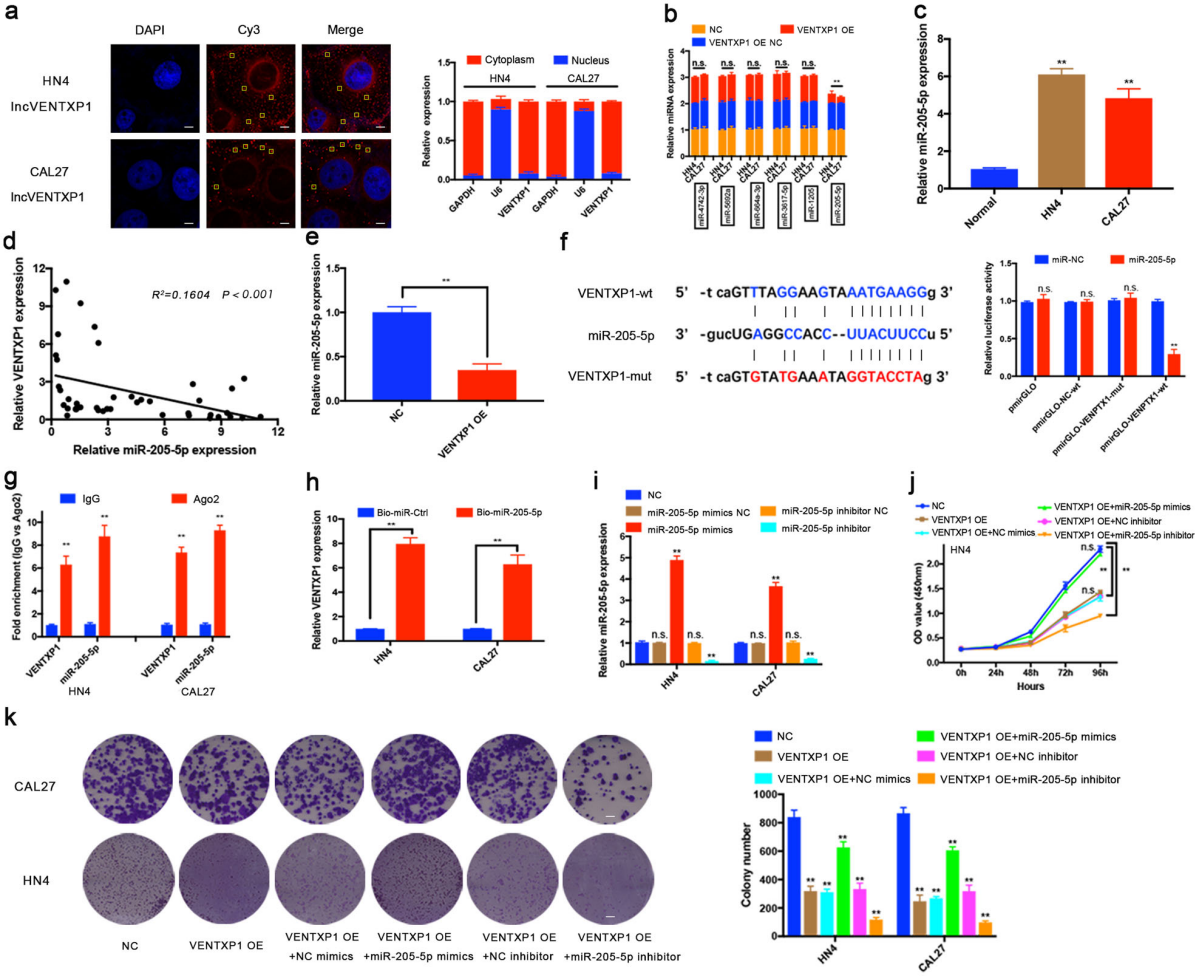
VENTXP1 acts as a sponge for miR-205-5p in the cytoplasm. **a** The distribution of VENTXP1 was analyzed in HN4 and CAL27 cells via FISH (left). The subcellular distribution of VENTXP1 in head and neck squamous cell carcinoma (HNSCC) cell lines was analyzed via qRT-PCR (right) (bar = 10 μm). **b** Relative expression levels of possible target miRNAs in VENTXP1-OE NC- or OE-transfected HN4 and CAL27 cells. **c** Expression levels of miR-205-5p were measured in HNSCC cell lines and normal oral keratinocytes (titled “Normal”). **d** The correlation between the VENTXP1 transcript level and the miR-205-5p RNA level was measured in 44 HNSCC tissues. **e** Relative expression levels of miR-205-5p in xenograft tumors. **f** Potential VENTXP1 base-pairing alignment with miR-205-5p (blue), as identified by starBase v2.0. VENTXP1 with a mutation in the putative binding site (red). Luciferase activity in CAL27 cells co-transfected with miR-205-5p mimics and luciferase reporters containing empty vector, VENTXP1 or mutant transcripts. **g** Associations of miR-205-5p and VENTXP1 with Ago2. HN4 and CAL27 cell lysates were collected for RIP with an anti-Ago2 antibody. Detection of miR-205-5p and VENTXP1 was performed via qRT-PCR. **h** Pull-down assay for biotin-labeled miRNA was used to evaluate the binding properties between miR-205-5p and VENTXP1 in two HNSCC cells, HN4 and CAL27. **i** Changes in the expression level of miR-205-5p in miR-NC-, miR-205-5p mimic-NC-, miR-205-5p mimics-, miR-205-5p inhibitor-NC-, and miR-205-5p inhibitor-transfected HN4 and CAL27 cells. **j**, **k** Growth and viability of VENTXP1 NC-, VENTXP1-OE, VENTXP1-OE + miR-205-5p mimics-NC-, VENTXP1-OE + miR-205-5p mimics-, VENTXP1-OE + miR-205-5p inhibitor-NC-, and VENTXP1-OE + miR-205-5p inhibitor-transfected HN4 and CAL27 cells were evaluated by CCK-8 and colony-formation assays (bar = 100 μm). The yellow square indicates an lncRNA VENTXP1 molecule. **P < 0.05; n.s. not significant.

The cellular localization of lncRNAs plays a critical role in function investigation. First, we examined the localization of VENTXP1 in HN4 and CAL27 cells. As shown in Fig. 4a, VENTXP1 was mainly located in the cytoplasm according to FISH and qRT-PCR assays. Previous studies have reported that lncRNAs serve as competing endogenous RNAs (ceRNAs) to sponge miRNAs when lncRNAs locate in the cytoplasm, thereby regulating proliferation and metastasis in cancer^20,21^. Given that VENTXP1 was enriched in the cytoplasm, we further explored whether VENTXP1 may serve as a platform for the RNA-induced silencing complex (RISC) catalytic subunit Argonaute 2 (Ago2) and acts as a ceRNA during the proliferation process of HNSCC cells.

To determine potential miRNAs that interact with VENTXP1, we used starBase, an online public resource that identifies miRNA target mRNAs and lncRNAs, which yielded several miRNA candidates. Next, we assessed the expression of these candidate miRNAs in VENTXP1-overexpressing HNSCC cells, and found that only miR-205-5p was significantly decreased after VENTXP1 overexpression (Fig. 4b). Similar results were obtained in VENTXP1-OE xenografts (Fig. 4e). Consistent with this finding, the expression levels of miR-205-5p were inversely correlated to VENTXP1 expression in both HNSCC cell lines and patient samples (Fig. 4c, d). Based on the predicted binding sites from starBase (Fig. 4f), we further investigated whether VENTXP1 binds to miR-205-5p to perform its function using a luciferase reporter construct carrying wild-type or mutated VENTXP1 sequences. This showed that miR-205-5p mimics reduced the luciferase activity of wild-type VENTXP1 reporters, not the luciferase activity from the VENTXP1 mutant (Fig. 4f). To further strengthen this finding, we performed a RIP assay with antibodies against Ago2, a crucial component of the RISC. As shown in Fig. 4g, both VENTXP1 and miR-205-5p were enriched following immunoprecipitation using the anti-Ago2 antibody compared to IgG. These data indicated that VENTXP1 may be recruited to Ago2-containing RISCs and functionally interacts with miR-205-5p in HNSCC cells. Furthermore, the pull-down assays revealed significantly higher VENTXP1 enrichment by biotin–miR-205-5p (Fig. 4h), confirming the direct interaction between the two molecules. In addition, cell-viability and colony-formation assay results showed that treatment with miR-205-5p inhibitor resulted in significantly impaired proliferation of CAL27 cells, while the miR-205-5p mimics restored the proliferation ability (Fig. 4i–k and Supplementary Fig. s2a). Collectively, these findings suggest that VENTXP1 can bind directly to miR-205-5p and both molecules play a role in regulating HNSCC proliferation.

### ANKRD2 is a downstream target of miR-205-5p and VENTXP1

**Fig. 5:**
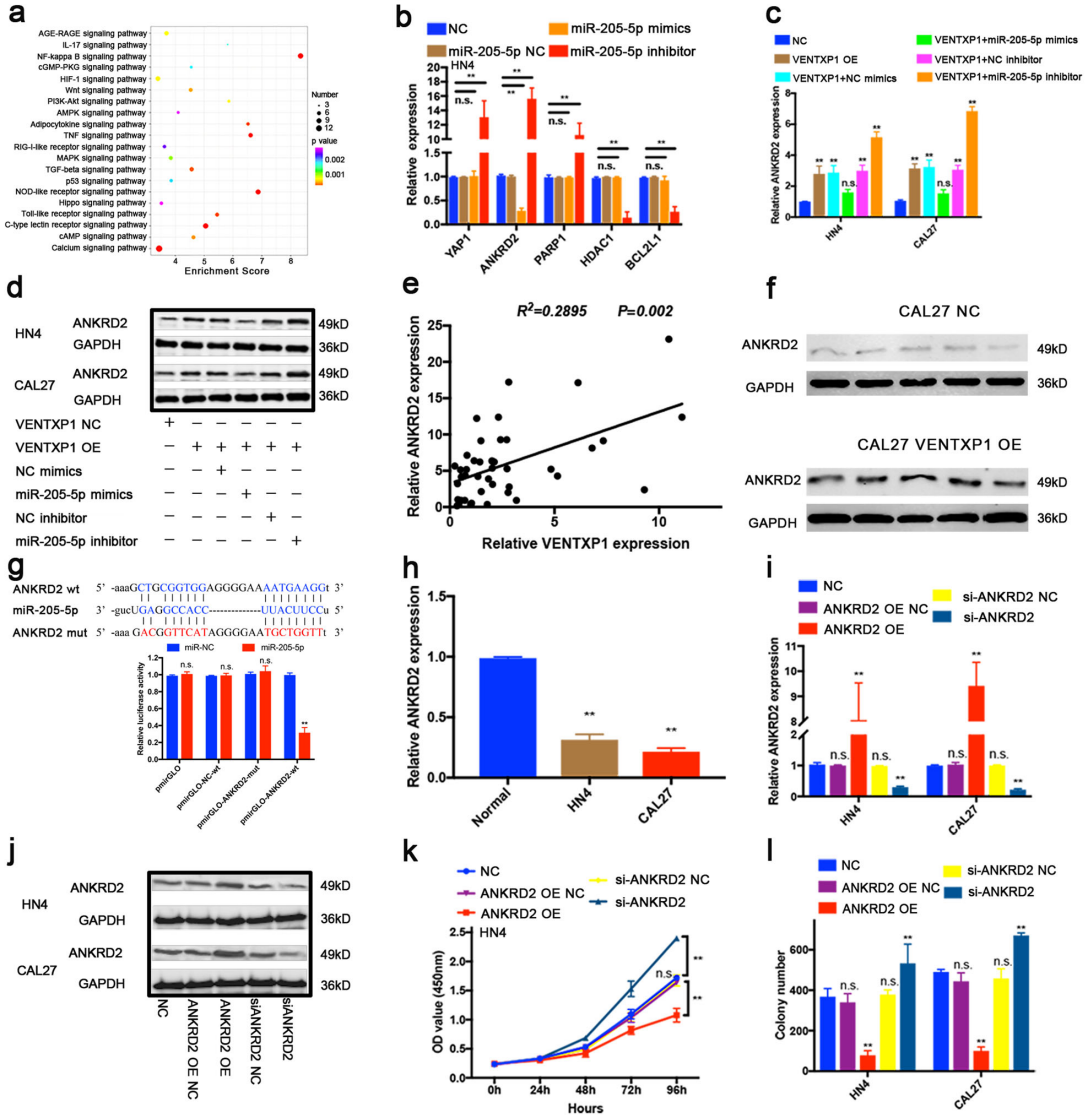
ANKRD2 is a downstream target of miR-205-5p and VENTXP1. **a** KEGG analysis to identify pathway terms enriched in the dysregulated mRNAs in miR-205-5p- knockdown head and neck squamous cell carcinoma (HNSCC) cells. **b** Relative expression of putative target genes in miR-205-5p-NC-, miR-205-5p mimics-, and miR-205-5p inhibitor-transfected HN4. **c**, **d** Expression of ANKRD2 in HN4 and CAL27 cells under different conditions was examined by qRT-PCR (**c**) and western blotting (**d**). **e** The correlation between the VENTXP1 transcript level and the ANKRD2 RNA level was measured in 44 HNSCC tissues. **f** Relative expression levels of ANKRD2 in xenograft tumors by western blotting. **g** Luciferase activity in CAL27 cells transfected with luciferase reporters containing ANKRD2-wt or mut. The data are represented as the relative ratios of firefly luciferase activity to Renilla luciferase activity. **h** QRT-PCR analysis of ANKRD2 levels in HN4 and CAL27 cells and normal oral primary keratinocytes (titled “Normal”). **i**, **j** Western blotting and qRT-PCR analyses of ANKRD2 expression in HN4 and CAL27 cells after treatment with ANKRD2 siRNAs or OE vector. **k** The viability of HN4 cells after transfection with ANKRD2-OE or siRNA was determined using CCK-8 assays. **l** Colony-formation assays were performed with HN4 and CAL27 cells after transfection with ANKRD2-OE or siRNA. **P < 0.05; n.s. not significant.

To elucidate the molecular mechanism underlying the contribution of miR-205-5p to tumorigenesis, we performed RNA-seq of CAL27 cells with stable depletion of miR-205-5p and identified thousands of genes with significant expression changes compared with parental CAL27 cells. GO and KEGG pathway analyses showed that several pathways were significantly associated with the biological processes of growth and death, supporting the role of miR-205-5p in HNSCC proliferation (Supplementary Fig. s3a, s3b). Among these, NF-kappa B signaling pathway is the most enriched pathway (Fig. 5a). We further studied the genes involved in NF-kappa B signaling, and chose to focus on five such genes (YAP1, ANKRD2, PARP1, HDAC1, and BCL2L1) with most fold changes in the miR-205-5p perturbation RNA-seq experiment.

qRT-PCR analysis showed that only ANKRD2 was downregulated when HNSCC cells were transfected with miR-205-5p mimics and upregulated when transfected with miR-205-5p inhibitor (Fig. 5b and Supplementary Fig. s3c). We further assessed the expression of ANKRD2 in VENTXP1-overexpressing HNSCC cells. ANKRD2 expression downregulated by miR-205-5p overexpression was amplified and miR-205-5p inhibitor maximized ANKRD2 expression in VENTXP1-overexpressing cells as accessed by both qRT-PCR and western blot (Fig. 5c, d and Supplementary Fig. s3d). This positive correlation of VENTXP1 and ANKRD2 expression was further confirmed in HNSCC tumor samples, VENTXP1-overexpressed xenografts (Fig. 5e, f), and in HNSCC cell lines (Fig. 5h).

To explore whether the observed effects of miR-205-5p are dependent on the regulation of 3′- untranslated regions (3′-UTR) of ANKRD2, we constructed a luciferase reporter vector containing the ANKRD2 3′-UTR with a wild-type or mutated miR-205-5p-binding site. The luciferase activity of the ANKRD2 3′-UTR wild-type reporter was significantly reduced in HNSCC cells transfected with the miR-205-5p mimics (Fig. 5g). However, no significant difference in luciferase activity was noted when the scrambled control or miR-205-5p mimics were co-transfected with the mutated ANKRD2 3′-UTR reporter. Thus, we concluded that ANKRD2 can be directly regulated by miR-205-5p.

### VENTXP1 inhibits HNSCC cell proliferation via regulation of the NF-kB signaling pathway

To elucidate the role of ANKRD2 in HNSCC progression, we overexpressed ANKRD2 using a pcDNA plasmid vector and downregulated ANKRD2 with siRNA in HN4 and CAL27 cells. The overexpression and knockdown efficiency were confirmed by western blotting and qRT-PCR 48 h later after transfection (Fig. 5I, j). Overexpression of ANKRD2 significantly reduced the cell viability and cell proliferation in both HN4 and CAL27 cell lines compared with the corresponding control cells. In contrast, knockdown of ANKRD2 significantly enhanced the cell viability and cell proliferation in both HN4 and CAL27 cell lines (Fig. 5k, l and Supplementary Fig. s3e, s3f).

**Fig. 6:**
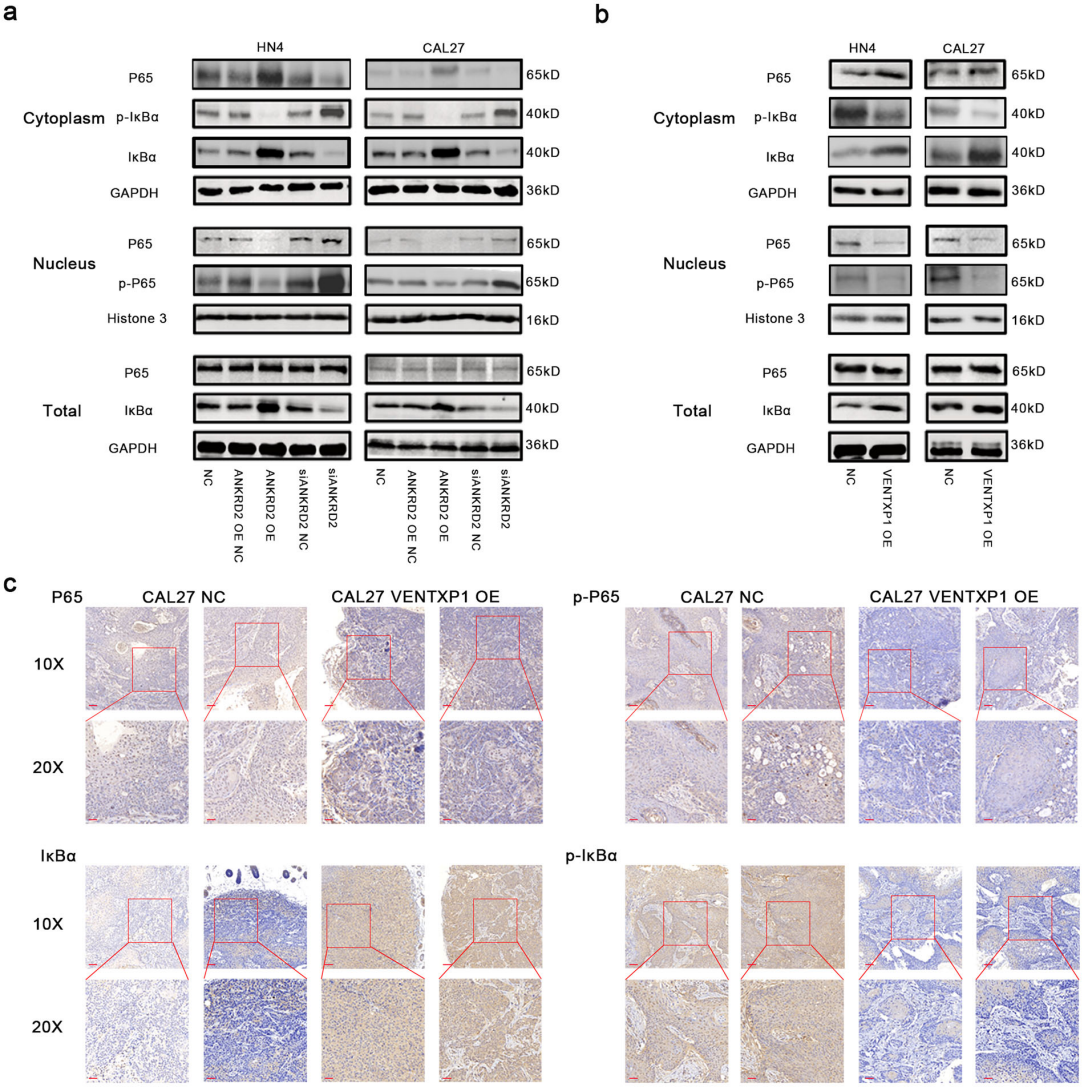
ANKRD2 modulates NF-kB-mediated tumor proliferation in head and neck squamous cell carcinoma (HNSCC). **a** Western blotting analysis of NF-kB signaling-related protein and phosphorylation levels in lysates from HN4 and CAL27 cells transfected with NC, ANKRD2-OE vector, or siRNA. **b** Western blotting analysis of NF-kB signaling-related protein and phosphorylation levels in lysates from HN4 and CAL27 cells transfected with NC or VENTXP1-OE vector. **c** The relative expression of key NF-kB signaling-related molecule staining was analyzed by immunohistochemistry in each group of tumors (bar = 100 μm, 50 μm, respectively).

Our previous results showed that NF-kappa B signaling pathway was most affected when miR-205-5p was knocked down (Fig. 5a), and some studies have reported ANKRD2-mediated reactivation of NF-kB signaling^22^. So we hypothesized that VENTXP1 and ANKRD2 may function to inhibit HNSCC cell proliferation via regulation of the NF-kB signaling pathway. To this end, we investigated the expression of NF-kB signaling pathway-related proteins (P65, phospho-P65, IkBα, and phospho-IkBα) after perturbation of ANKRD2 expression in HNSCC cells. ANKRD2 overexpression suppressed NF-kB signaling in HNSCC cells, while the opposite effects were observed in cells with ANKRD2 knockdown (Fig. 6a). In addition, overexpression of VENTXP1 showed similar effects on NF-kB signaling when compared to the results from ANKRD2-overexpressing cells (Fig. 6b). Then, we evaluated the expression of NF-kB signaling pathway-related proteins (P65, p-P65, IkBα, and p-IkBα) in mouse tumors by immunohistochemistry. The levels of p-P65 and p-IkBα were lower in VENTXP1-overexpressing tumors than in control tumors, whereas the IKBα level was higher. While the overall level of P65 was almost unchanged, the level of P65 in the cytoplasm was higher, and in the nucleus, it was lower (Fig. 6c). Collectively, these data imply that VENTXP1 can inhibit HNSCC cell proliferation through regulation of the ANKRD2/NF-kB signaling pathway both in vitro and in vivo.

## Discussion

Recent studies in genome and transcriptome have identified numerous cancer-related lncRNAs^5^. In our study, we integrated multidimensional genomic and epigenetic data from four types of tumor samples and cancer cell lines in the TCGA and CCLE databases. Our analysis demonstrated that lncRNAs can be epigenetically silenced via DNA methylation in the promoter region. We hypothesize that lncRNAs targeted for epigenetic alteration in cancer may play an important role in tumor proliferation and metastasis. In addition, epigenetically regulated lncRNAs identified in our study include numerous cancer-related lncRNAs, such as Linc02273^23^, MEG3^24^, HOTAIR^9^, and AFAP1-AS^25^.

The human transcriptome is estimated to contain approximately 20,000 lncRNAs. Although some lncRNA transcripts may represent transcriptional noise, lncRNAs have critical and broad regulatory roles in biological activities, including post-transcriptional regulation, organization of protein complexes, cell–cell signaling, and allosteric regulation of proteins^26^. Dysregulation of gene expression, including protein-coding and non-protein-coding genes, is critical for carcinogenesis and metastasis^27,28^.

Encouraged by the recapitulation of documented cancer-related lncRNAs, we validated the frequently epigenetically silenced lncRNA, VENTXP1, as a putative tumor suppressor. The lncRNA VENTXP1 has never been reported to inhibit tumor proliferation in any solid tumor. Our present study showed that VENTXP1 was weakly expressed in HNSCC biopsies, and Kaplan–Meier (K–M) survival analysis demonstrated that VENTXP1 expression was negatively correlated with poor prognosis. Furthermore, with a lentiviral vector system, VENTXP1 was overexpressed in HNSCC cells. Increased expression of VENTXP1 inhibited the HNSCC cell growth, proliferation, and colony formation. These findings indicate that VENTXP1 may have a tumor-suppressing function in HNSCC and it is a promising novel diagnostic and prognostic biomarker for HNSCC. To our knowledge, this report is the first to reveal the association of VENTXP1 expression with the survival of HNSCC. However, the exact mechanisms through which VENTXP1 is involved in HNSCC require further investigation.

Recently, the lncRNA-associated ceRNA networks have been revealed to play an important role in human lung cancer^29^, renal carcinoma^30^, and human hepatocellular cancer^31^. Consistent with these findings, we showed that VENTXP1 could bind miR-205-5p and regulate the proliferation of HNSCC cells. MiR-205-5p has been reported to be involved in several solid tumors, such as breast cancer^32^, prostate carcinoma^33^, and hepatocellular cancer^34^. MiR-205-5p can repress the expression of endogenous E2F1 and regulate chemotherapeutic resistance^35^. In addition, miR-205-5p plays a vital role in mediating cisplatin resistance in nasopharyngeal carcinoma cells via the PI3K–Akt pathway^36^. Recently, studies showed that miR-205-5p promotes genomic instability in HNSCC via the BRCA1/RAD17 axis^37^. In our current study, we identified several miRNA for VENTXP1 through a public database, and RIP and luciferase assays confirmed that VENTXP1 might act as a sponge of miR-205-5p to inhibit the expression of certain miRNAs through directly binding to them in HNSCC cells. These observations suggested that VENTXP1 may regulate the proliferation of HNSCC cells through a ceRNA network via the VENTXP1/miR-205-5p axis.

NF-kB signaling plays important roles in multiple biological processes, including the immune response, differentiation, and cell survival, proliferation, and migration^38–41^. Our results showed that the NF-kappa B signaling pathway was the most enrichment-changed gene pathway when expression of miR-205-5p was changed in HNSCC cells, then we observed an inverse correlation between ANKRD2 mRNA or protein expression of ANKRD2 and the levels of miR-205-5p in HNSCC cell lines, and the luciferase assay results confirmed that ANKRD2 is a direct target of miR-205-5p. In addition, the expression levels of ANKRD2 were elevated and those of miR-125a-3p were decreased in VENTXP1-overexpressing HNSCC cell lines. Besides, several studies have verified that ANKRD2 can modulate NF-kB-mediated inflammatory responses^22,42^. According to the results, our study indicated that ANKRD2 could inhibit HNSCC proliferation by downregulating the NF-kB signaling pathway.

**Fig. 7:**
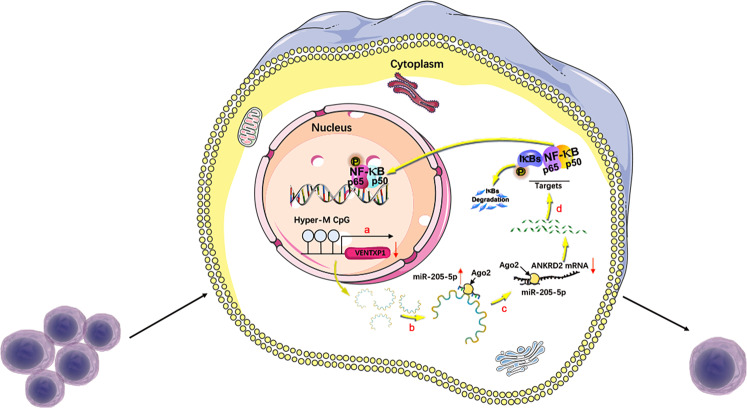
Hypothetical model describing the function of the lncRNA VENTXP1 function in head and neck squamous cell carcinoma (HNSCC). **a** Hypermethylation in VENTXP1 promoter inhibits the expression of VENTXP1. **b** VENTXP1 serves as a miRNA sponge of miR-205-5p. **c** Mi-205-5p participates in the regulation of ANKRD2 expression. **d** ANKRD2 regulates HNSCC proliferation by modulating NF-kB signaling.

In summary, our study identifies that VENTXP1 competitively sponges miR-205-5p to block the suppressive effect of miR-205-5p on ANKRD2, regulating NF-kB signaling and then inhibiting HNSCC proliferation (Fig. 7). These data provide novel insight into understanding the progression and therapy of HNSCC, and offer potential predictive and therapeutic strategies for HNSCC patients.

## Materials and methods

### Data collection

DNA methylation, whole-exome mutation, and GISTIC copy number alteration data were downloaded from TCGA database (https://gdac.broadinstitute.org/). LncRNA annotation data were downloaded from GENCODE website (https://www.gencodegenes.org/). DNA methylation and RNA-seq data for four types of cancer cell lines (HNSCC, liver, breast, and lung cancer) were downloaded from the CCLE database (https://portals.broadinstitute.org/ccle).

### Patients and tissue samples

Fresh HNSCC and adjacent normal tissues from 44 human papillomavirus (HPV)-negative patients (collected postoperatively between April 2010 and October 2014) were collected by the Department of Oral and Maxillofacial Head and Neck Oncology, Shanghai Ninth People’s Hospital. Patients were diagnosed by pathological examination. Tumors were classified according to the TNM staging system (2010 version). This study was approved by the Human Research Ethics Committee of the Ninth People’s Hospital, Shanghai JiaoTong University School of Medicine (Shanghai, China). The clinical patient data are shown in Table 1.

### Cell culture

The HNSCC cell lines HN4, HN6, and HN30 were obtained from the University of Maryland, USA. CAL27, SCC9 cells were purchased from the Type Culture Collection of the Chinese Academy of Sciences (Shanghai, China). All cell lines were verified by short tandem-repeat genotyping. Normal oral primary keratinocytes were cultured from gingival tissues of healthy patients after tooth extraction. SCC9 cells were maintained in Dulbecco’s modified Eagle’s medium (DMEM)/F12, and the other cell lines were maintained in DMEM (Gibco, Grand Island, NY, USA) supplemented with 10% fetal bovine serum, 1% glutamine, and 1% penicillin–streptomycin. Cells were cultured in a standard humidified atmosphere of 5% CO_2_ at 37 °C.

### Cell transfection

VENTXP1 siRNA, VENTXP1-overexpression vector, miR-205-5p mimics, control mimic (NC mimic), miR-205-5p inhibitor, control inhibitor (NC inhibitor), ANKRD2 siRNA, and overexpression vector were synthesized by GenePharma Co. (Shanghai, China). The miR-205-5p mimics and miR-205-5p inhibitor, and viral vector for VENTXP1 overexpression were also synthesized by GenePharma Co. (Shanghai, China). Cells were grown in 6-well plates and transfected using Lipofectamine 3000 according to the manufacturer’s instructions. Cells were collected 48 h after transfection for real-time PCR or Western blotting analyses.

### Genomic DNA isolation and bisulfite-sequencing PCR

Genomic DNA was extracted from HNSCC cells with a TIANamp genomic DNA kit (TIANGEN). Bisulfite-sequencing PCR was performed with Oebiotech reagents. The sequencing primers were F: 5′-GGTTTTTGTTAGTTATTTTGAAAAAGG-3′ and R: 5′-TCAATCCATAAAACCAAACCTAAA-3′.

### Statistical analysis

All statistical analyses were performed using SPSS 20.0 (SPSS, Chicago, IL, USA). GraphPad Prism 6.0 (GraphPad Software, San Diego, CA, USA) was used for plotting and graphics. The expression differences between HNSCC and matched normal tissues were analyzed using paired samples *t* tests. Pearson’s coefficients were calculated for expression correlation analyses. The expression differences between high/low stages, the expression changes after transfection, and the cell proliferation assay results were analyzed using independent-sample *t* tests. *P* values were two-sided, and a value of 0.05 was considered significant (**P < 0.05). All values are expressed as the means ± standard errors.

### Supplementary methods

RNA extraction, reverse transcription (RT), quantitative PCR (qPCR), cytoplasmic and nuclear RNA isolation, cell proliferation assay, colony-formation assays, immunohistochemistry, fluorescence in situ hybridization (FISH), RNA immunoprecipitation (RIP) assay, luciferase reporter assay, pull-down assay with biotinylated miRNA, western blotting, and xenograft mouse model are described in Supplementary File 1.

## Supplementary information

supplementary fig 1

supplementary fig 2

supplementary fig 3

Supplementary fig information.

supplementary file 1

supplementary file 2
