# Supplementary figures and images for: Epigenetic regulation of VENTXP1 suppresses tumor proliferation via miR-205-5p/ANKRD2/NF-kB signaling in head and neck squamous cell carcinoma
Source: Cell Death Dis. 2020 Oct 9;11(10):838. doi: 10.1038/s41419-020-03057-w (PMC7547684; doi:10.1038/s41419-020-03057-w)

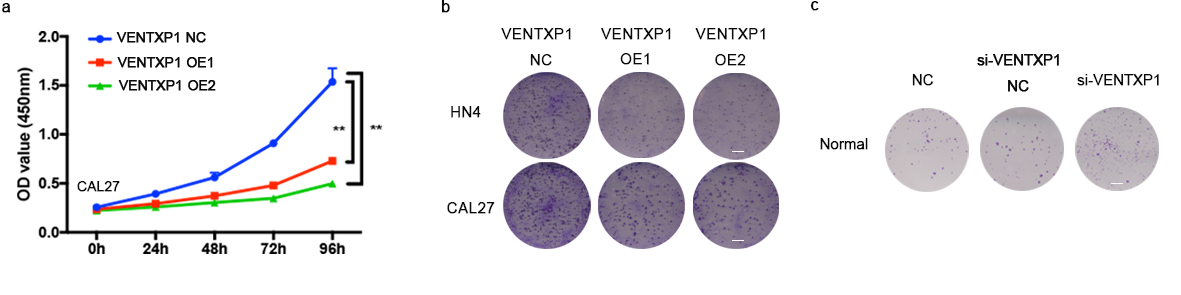

Supplement: Supplementary file 1 — supplementary fig 1 [file 41419_2020_3057_MOESM1_ESM.tif]

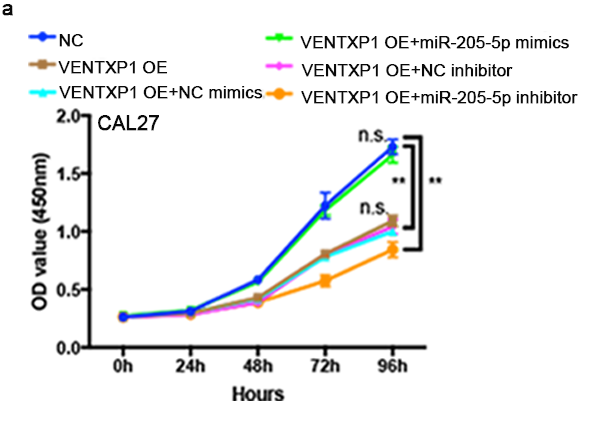

Supplement: Supplementary file 2 — supplementary fig 2 [file 41419_2020_3057_MOESM2_ESM.tif]

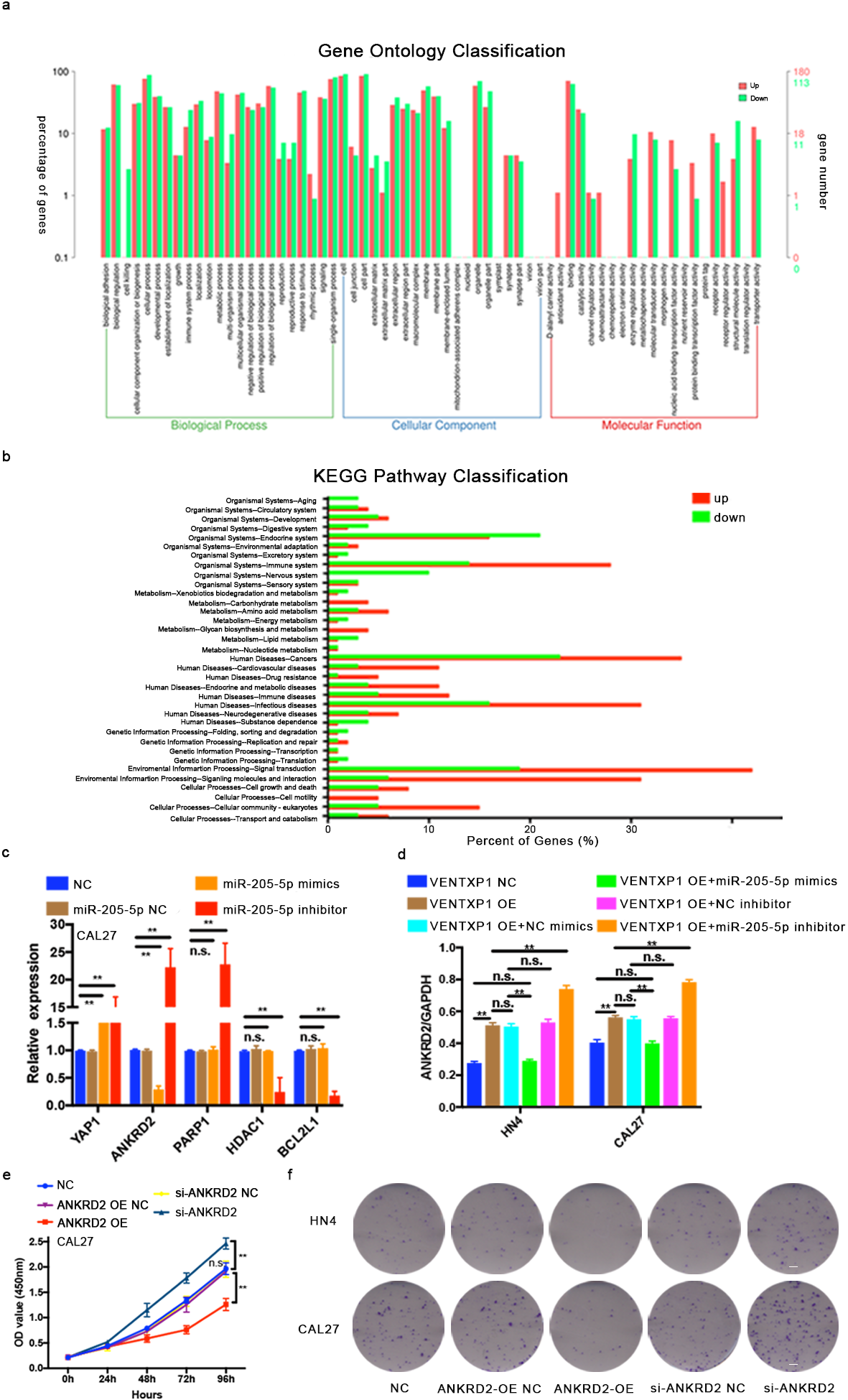

Supplement: Supplementary file 3 — supplementary fig 3 [file 41419_2020_3057_MOESM3_ESM.tif]
