# Supplementary material for: Epigenetic regulation of VENTXP1 suppresses tumor proliferation via miR-205-5p/ANKRD2/NF-kB signaling in head and neck squamous cell carcinoma
Source: Cell Death Dis. 2020 Oct 9;11(10):838. doi: 10.1038/s41419-020-03057-w (PMC7547684; doi:10.1038/s41419-020-03057-w)
Supplement: Supplementary file 4 — Supplementary fig information. [file 41419_2020_3057_MOESM4_ESM.docx]

**Supplementary Information: legends of supplementary figures (Fig s1–3).**

**Supplementary Fig s1: (a)** Cell growth viability was measured in VENTXP1-OE (overexpression plasmid)-transfected CAL27 cells by CCK-8 assays. **(b)** Representative results of colony formation assays after HN4 and CAL27 cells were transfected with VENTXP1-NC or -OE. (bar = 100 μm). **(c)** Representative results of colony formation assays after normal oral primary keratinocytes were transfected with si-VENTXP1 NC or si-VENTXP1. (bar = 100 μm).

**Supplementary Fig s2: (a)** Growth and viability of VENTXP1 NC-, VENTXP1-OE, VENTXP1-OE + miR-205-5p mimics-NC-, VENTXP1-OE + miR-205-5p mimics-, VENTXP1-OE + miR-205-5p inhibitor-NC-, and VENTXP1-OE + miR-205-5p inhibitor-transfected CAL27 cells were evaluated by CCK-8 assays.

**Supplementary Fig s3: (a)** GO term enrichment analysis of the biological process (BP), cellular component (CC) and molecular function (MF) categories for the dysregulated mRNAs. **(b)** KEGG analysis to identify Pathway terms enriched in the dysregulated mRNAs in miR-205-5p knockdown HNSCC cells. **(c)** Relative expression of changed target genes in miR-205-5p-NC-, miR-205-5p mimics-, miR-205-5p inhibitor-transfected CAL27. **(d)** Expression of ANKRD2 in VENTXP1 NC-, VENTXP1-OE, VENTXP1-OE + miR-205-5p mimics-NC-, VENTXP1-OE + miR-205-5p mimics-, VENTXP1-OE + miR-205-5p inhibitor-NC-, VENTXP1-OE + miR-205-5p inhibitor-transfected HN4 and CAL27 cells were confirmed by western blotting. **(e)** The viability of CAL27 cells after transfection with ANKRD2-OE or siRNA was determined by using CCK8 assays. **(f)** Colony formation assays were performed with HN4 and CAL27 cells after transfection with ANKRD2-OE or siRNA. (bar = 100 μm).
