# Supplementary material for: Epigenetic regulation of VENTXP1 suppresses tumor proliferation via miR-205-5p/ANKRD2/NF-kB signaling in head and neck squamous cell carcinoma
Source: Cell Death Dis. 2020 Oct 9;11(10):838. doi: 10.1038/s41419-020-03057-w (PMC7547684; doi:10.1038/s41419-020-03057-w)
Supplement: Supplementary file 5 — supplementary file 1 [file 41419_2020_3057_MOESM5_ESM.docx]

**Supplementary methods**

**RNA extraction, reverse transcription (RT) and quantitative PCR (qPCR)**

Total RNA from tissue specimens and cells was extracted using TRIzol reagent (Invitrogen, USA). The RNA concentration and integrity were determined by spectrophotometry and standard RNA gel electrophoresis. RNA was reverse transcribed into cDNAs using a Primer-Script One Step RT-PCR kit (TaKaRa, Dalian, China). The cDNA template was amplified by real-time PCR using a SYBR Premix DimerEraser kit (TaKaRa). The primer sequences were used for PCR are listed in Supplementary file 2. All PCRs were performed in triplicate. mRNA expression levels were normalized to those of actin.

Real-time PCRs were performed in triplicate in an ABI7300 system (Applied Biosystems, Carlsbad, CA, USA). Gene expression in each sample was normalized to actin expression. The relative expression fold changes in mRNA expression were calculated by the 2^−ΔΔCt^ method.

**Cytoplasmic and nuclear RNA isolation**

Cytoplasmic and nuclear RNA were isolated using an RNA Purification Kit (Norgen Biotek, Thorold, ON, Canada) according to the manufacturer’s instructions. GAPDH was used to normalize cytoplasmic mRNA expression levels, and U6 was used to normalize nuclear mRNA expression levels. The relative fold changes in mRNA expression were calculated by the 2^−ΔΔCt^ method.

**Cell proliferation assay**

Cellular proliferation was measured 24 h after transfection with a Cell Counting Kit-8 (CCK-8, Dojindo Laboratories, Kumamoto, Japan) based on the manufacturer’s instructions. CAL27 and HN4 cells were seeded into 96-well plates at a density of 1 × 10^3^ cells per well in 100 μl of medium and incubated at 37 °C. At the indicated time points, 10 μl of CCK-8 solution was added to each well. After incubation at 37 °C for 1 h, the absorbance at 450 nm was measured with a plate reader. The growth curves were examined to determine the growth rates.

**Colony formation assays**

For the colony formation assay, a total of 800 cells plated in a clean six-well plate and maintained in medium containing 10% FBS. The medium was replaced every 3 days. After 14 days, the cells were fixed with methanol and stained with 0.1% crystal violet (Sigma-Aldrich, St. Louis, MO, USA). Visible colonies were manually counted. Triplicate wells were established for each treatment group.

**Immunohistochemistry**

Immunohistochemistry was performed on paraformaldehyde-fixed paraffin sections. Ki-67, P65, p-P65, ikbα (Cell Signaling Technology), and p-ikbα antibodies (Santa Cruz) were used to perform immunohistochemistry via the streptavidin-peroxidase conjugate method. The percentage of positive tumor cells was graded according to the following criteria: 0, < 10% positive; 1, 10-30% positive; 2, 31-50% positive; and 3, > 50% positive. HNSCC tissues with different staining levels were divided into the low-expression group (staining scores of 0 or 1) and the high-expression group (staining scores of 2 or 3).

**Fluorescence in situ hybridization (FISH)**

FISH was conducted according to the manufacturer’s instructions (RiboBio Company, Guangzhou, China). In brief, a lncRNA VENTXP1-specific probe was designed, synthesized. 5 x 10^4^ cells were seeded on a glass-bottomed confocal plate and cultured overnight. After fixation with 4% PFA and permeabilization with 0.5% Triton, hybridization was carried out overnight with the probes conjugated with Cy3 at 37°C in 2×SSC, 10% formamide and 10% dextran. Finally, the nuclei were stained by DAPI and the images were captured under a confocal microscope.

**RNA immunoprecipitation (RIP) assay**

RNA immunoprecipitation (RIP) assays were performed according to the instructions of the Millipore Magna RIP Kit (Millipore, Darmstadt, Germany). Cells were lysed in complete RIP lysis buffer. Then, 100μl of cell lysate was incubated with RIP buffer containing magnetic beads conjugated to a human anti-Ago2 antibody (1:50 dilution, Millipore) and a negative control antibody (normal mouse IgG). The samples were then incubated with proteinase K to isolate immunoprecipitated RNA. The isolated RNA was analyzed via qRT-PCR, which was performed using SYBR Green (TaKaRa, Dalian, China) with primers.

**Luciferase reporter assay**

PmirGLO, pmirGLO-VENTXP1-wt or pmirGLO-VENTXP1-mut was cotransfected with the miRNA-205-5p mimics or NC-mimic into VENTXP1-overexpressing CAL27 cells in a 6-well dish by using Lipofectamine 2000 reagent (Invitrogen, USA) according to the manufacturer's instructions. Similarly, pmirGLO, pmirGLO-ANKRD2-wt or pmirGLO-ANKRD2-mut was transfected into VENTXP1-overexpressing CAL27 cells by using Lipofectamine 2000 reagent (Invitrogen, USA) according to the manufacturer's instructions. The relative luciferase activity was normalized to Renilla luciferase activity 48 h after transfection. The cells were cotransfected with the pRenilla Renilla luciferase reporter to normalize for transfection efficiency. Six hours after transfection, the transfection medium was replaced with fresh medium, and the cells were cultured for another 24 h. The cells were pretreated with the external stimulant for 12 h and harvested in passive lysis buffer. Finally, luciferase activity was measured using a Dual Luciferase System (Promega, USA).

**Pull-down assay with biotinylated miRNA**

The biotinylated 3′ end of miR-205-5p mimics or control RNA (GenePharma Co.) was transfected into HN4 and CAL27 cells at a final concentration of 100 nM. Two days after transfection, whole cell lysates were harvested. The biotinylated RNA complex was pulled down by incubating the cell lysates with streptavidin-coated magnetic beads at 4 °C on the rotator overnight. TRIzol reagent (Invitrogen) was used to extract RNA from the input and pull-down beads. The abundance of VENTXP1 in the bound fraction was evaluated by qRT-PCR analysis.

**Western blotting**

Western blotting was performed as described in our previous study. GAPDH was used as a control. Histone H3 was used as a control for nuclear protein. Antibodies (1:1000) specific for ANKRD2, P65, p-P65, Ikbα, p-ikbα and histone H3 were purchased from Cell Signaling Technology (Boston, MA, USA).

**Xenograft mouse model**

BALB/c nude mice (aged 4~6 weeks) purchased from the Shanghai Laboratory Animal Center (Shanghai, China) were bred in specific pathogen-free (SPF) facilities. CAL27 cells (1 × 10^6^) stably expressing LV-NC or overexpressing LV-VENTXP1 (OE) were subcutaneously injected into either side of the flank area of 6-week-old female athymic nude mice (n=8 mice per group). Tumor volumes were measured (0.5 × length × width^2^) in the mice every four days and tumors were finally weighed after the mice were sacrificed. After 5 weeks, the mice were sacrificed, and the excised tumors were fixed and embedded in paraffin for immunohistochemical staining analysis of Ki67, P65, p-P65, ikbα, and p-ikbα. All animal experiments were performed in the animal laboratory center of the Ninth People's Hospital, Shanghai JiaoTong University School of Medicine (Shanghai, China) and were conducted in accordance with the Guide for the Care and Use of Laboratory Animals published by the US National Institutes of Health (NIH publication number 85-23, revised 1996). The study protocol was approved by the Animal Care and Use Committee of the Ninth People's Hospital.
