# Supplementary material for: Epigenetic regulation of VENTXP1 suppresses tumor proliferation via miR-205-5p/ANKRD2/NF-kB signaling in head and neck squamous cell carcinoma
Source: Cell Death Dis. 2020 Oct 9;11(10):838. doi: 10.1038/s41419-020-03057-w (PMC7547684; doi:10.1038/s41419-020-03057-w)
Supplement: Supplementary file 6 — supplementary file 2 [file 41419_2020_3057_MOESM6_ESM.docx]

**Table S1. Primers of genes used for this study.**

| Primer | Sequences |
| --- | --- |
| LINC00461 | Forward Primer 5’- GACATTTACGCCACAACCCACG -3’  Reverse Primer 5’- AGACAGACCCTCAGATTCCCCA -3’ |
| LINC00668 | Forward Primer 5’- GCACC TGTCATTGCAATTCC -3’  Reverse Primer 5’- CTGTGTCTAACTTAGTGGCTC -3’ |
| VENTXP1 | Forward Primer 5’- CGGTCTGTGCAGCTAACACCA -3’  Reverse Primer 5’- TCCAGAACCTTTGCGTGGGT -3’ |
| RP11-81H3.2 | Forward Primer 5’- CCGGATGCCAGTCTACTACG -3’  Reverse Primer 5’- TGATGTGCCAGGGAAGAAAGCCTA -3’ |
| Actin | Forward Primer 5’- CATGTACGTTGCTATCCAGGC -3’  Reverse Primer 5’- CTCCTTAATGTCACGCACGAT -3’ |
| GAPDH | Forward Primer 5’- GGAGCGAGATCCCTCCAAAAT -3’  Reverse Primer 5’- GGCTGTTGTCATACTTCTCATGG -3’ |
| U6 | Forward Primer 5’- CTCGCTTCGGCAGCACATATACT -3’  Reverse Primer 5’- ATTTGCGTGTCATCCTTGCGCA -3’ |
| ANKRD2 | Forward Primer 5’- ATTGAGAAGTTCCTGGCTGACG -3’  Reverse Primer 5’- AAGCTCGGTGCAGTGCTGTC -3’ |
| YAP1 | Forward Primer 5’- TAGCCCTGCGTAGCCAGTTA-3’  Reverse Primer 5’- TCATGCTTAGTCCACTGTCTGT -3’ |
| PARP1 | Forward Primer 5’- TGGAAAAGTCCCACACTGGTA -3’  Reverse Primer 5’- AAGCTCAGAGAACCCATCCAC -3’ |
| HDAC1 | Forward Primer 5’- CTACTACGACGGGGATGTTGG -3’  Reverse Primer 5’- GAGTCATGCGGATTCGGTGAG -3’ |
| BCL2L1 | Forward Primer 5’- GAGCTGGTGGTTGACTTTCTC -3’  Reverse Primer 5’- TCCATCTCCGATTCAGTCCCT -3’ |
